# The Vasohibin Family

**DOI:** 10.3390/ph3020433

**Published:** 2010-02-05

**Authors:** Yasufumi Sato

**Affiliations:** Department of Vascular Biology, Institute of Development, Aging and Cancer, Tohoku University, 4-1 Seiryo-machi, Aoba-ku, Sendai 980-8575, Japan; E-Mail: y-sato@idac.tohoku.ac.jp; Tel.: +81-22-717-8528; Fax: +81-22-717-8533

**Keywords:** endothelial cell, negative feedback, mononuclear cell, bone marrow

## Abstract

Angiogenesis is regulated by the local balance between angiogenesis stimulators and inhibitors. A number of endogenous angiogenesis inhibitors have been found in the body. The origin of these inhibitors is mostly extrinsic to the vasculature. Recently, however, vascular endothelial cells themselves have been found to produce angiogenesis inhibitors including vasohibin-1. These intrinsic inhibitors are thought to regulate angiogenesis by an auto-regulatory or negative-feedback mechanism. This review will focus on vasohibin-1 produced by vascular endothelial cells and on its homologue, vasohibin-2.

## 1. Introduction

The vasculature is primarily composed of luminal endothelial cells (ECs) and surrounding mural cells (smooth muscle cells or pericytes). Coverage by mural cells stabilizes blood vessels. The initial step of angiogenesis is the extrication of mural cells from endothelial tubes for vascular destabilization. Subsequently, specialized ECs, the so-called “tip” cells, migrate by extending numerous filopodia, whereas following ECs, the so-called “stalk” cells, proliferate, causing elongation of the sprouts to form immature tube-like structures. Finally, redistributed mural cells affix themselves to the newly formed vessels for vascular restabilization. By this process, ECs stop their proliferation, thus terminating angiogenesis [1].

The body contains a number of endogenous angiogenesis stimulators and inhibitors, and the local balance between them regulates this process of blood vessel formation. Angiogenesis stimulators are mostly growth factors and cytokines including vascular endothelial growth factor (VEGF), whereas angiogenesis inhibitors are variable and include hormones, chemokines, proteins accumulated in the extracellular matrix, proteolytic fragments of various proteins, and so forth [2]. In addition, the majority of angiogenesis inhibitors are extrinsic to the vasculature; some are constitutively expressed and act as barriers to prevent the invasion of sprouts, and the others are generated in response to stimuli and counteract this process. Recently, however, ECs themselves have been found to produce intrinsic angiogenesis inhibitors. Such inhibitors may regulate angiogenesis in an auto-regulatory or negative-feedback fashion. This review will focus on one of such factors vasohibin-1 (VASH1) and its homologue vasohibin-2 (VASH2). VASH2 is closely related to VASH1, but is mostly extrinsic to endothelium.

## 2. Vasohibin-1 (VASH1)

We hypothesized that ECs themselves might produce either positive of negative regulators of angiogenesis. To test this hypothesis, we performed DNA microarray analysis to detect VEGF-inducible genes in ECs [3]. Among a variety of VEGF-inducible genes, we focused our attention on those genes whose functions were undefined at that time. We performed *in vitro* functional assays for angiogenesis, isolated the molecule VASH1, and demonstrated its anti-angiogenic activity *in vivo* [4]. 

The gene for human *VASH1* gene is located on chromosome 14q24.3, and consists of 8 exons and 7 introns. Human VASH1 protein is composed of 365 amino acid residues without any glycosylation sites. A cluster of basic amino acids is present in the C-terminus region, but neither a classical secretion signal sequence nor any other functional motifs are found in its entire amino acid sequence. Since VASH1 protein is present in medium conditioned by ECs, the lack of the classical signal sequence suggests that VASH1 is an unconventional secretory protein [4]. Mouse VASH1 is more than 90% identical to its human counterpart in terms of amino acid sequence, indicating that *VASH1* is a highly conserved gene at least between humans and mice (Table 1). One alternative splicing form of VASH1 lacking exons 5 to 8 has been reported to exist in humans [4,5]. This splicing variant also maintains anti-angiogenic activity [6].

**Table 1:** Locus and similarity between human and mouse vasohibins.

	Locus	hVASH1	mVASH1	hVASH2	mVASH2
hVASH1	14q24.3	100%	91.2%	52.5%	51.9%
mVASH1	12D2		100%	50.9%	50.9%
hVASH2	1q32.3			100%	97.5%
mVASH2	1H6				100%

The expression of VASH1 in ECs is induced not only by VEGF but also by fibroblast growth factor 2 (FGF-2), another potent angiogenic factor [4,5]. The intracellular signaling for the induction of VASH1 by VEGF was characterized by using blocking anti-VEGFRs mAbs to test which receptor was involved in the induction of VASH1. It appears that anti-VEGFR-2 antibodies but not anti-VEGFR-1 antibodies inhibited the VEGF-stimulated induction of VASH1 [5]. The downstream intracellular signaling pathways of VEGFR-2 for the induction of VASH1 have been further investigated. GF109203X, a broad-spectrum inhibitor of protein kinase C (PKC), strongly inhibits the increase in VASH1 mRNA and protein in response to VEGF, which finding is in line with the observation that phorbol 12-myristate 13-acetate (PMA), an activator of PKC, enhances the expression of VASH1. Selective PKC isoform inhibitors were used to clarify which PKC isoforms are involved in the up-regulation of VASH1. Rottlerin, a specific inhibitor of PKC, completely blocks the up-regulation of VASH1, whereas Gö6976, a specific inhibitor of PKC, and HBDDE, an inhibitor of PKC and PKC, only partially inhibit it. Hispidin, a specific inhibitor of PKC, does not affect the up-regulation of VASH1. From these results, we concluded that PKC transduce a sprincipal signal for the up-regulation of VASH1 through VEGF [5]. FGF-2 increases the expression of VASH1 in ECs to a level comparable to that obtained with VEGF, and rottlerin again completely blocks FGF-2-stimulated up-regulation of VASH1 [5]. Accordingly, the principal signaling pathways for the induction of VASH1 by these two representative angiogenic growth factors considerably overlap. Actinnomycin D treatment does not change the decay of VEGF-induced VASH1 mRNA [5], and thus the increase in VASH1 mRNA by VEGF is not determined by mRNA stability. However, when cycloheximide is added, the expression of VASH1 mRNA is completely abolished under both basal and VEGF-stimulated conditions [5]. Therefore, *de novo* protein synthesis is indispensable for the induction of VASH1 mRNA.

The calculated molecular weight of the VASH1 protein is 44 kDa. However, Western blotting shows the presence of multiple bands of VASH1 [7]. So we evaluated the possibility of posttranslational modification of the VASH1 protein. When we overexpressed VASH1 cDNA in a HUVEC-derived cell line, we detected at least 4 bands (42, 36, 32, and 27 kDa) by Western blotting. In order to characterize the structures of these multiple forms of VASH1 proteins, various VASH1 cDNA mutants were generated to substitute some basic amino acids. Since the complete 44 kDa form was absent, the amino terminal region is thought to be processed simultaneously or immediately after the translation. We also determined two cleavage sites in the amino terminal region, *i.e.*, arginine 29 and arginine 76. The 42 kDa form is generated by the cleavage at arginine 29, whereas the 36 kDa form is generated by the cleavage at arginine 76. Cleavage sites in the carboxyl terminal region have not been determined yet. However, since the calculated molecular weight of the VASH1 protein from its methionine 77 to the carboxyl terminal end is 33 kDa, the carboxyl terminal of the 32 kDa form should be very close to the end. From the calculation of the molecular weight, the 27 kDa form may lack about 47 amino acids from the carboxyl terminal, which region contains the cluster of basic amino acids [7].

Immunohistochemical analysis revealed that VASH1 protein is present in ECs in the developing human or mouse embryo, but is reduced in expression in the post-neonate [8]. Nimmagadda *et al*. independently showed by *in situ* hybridization that VASH1 mRNA is expressed in a wide range of tissues and organs in the chicken embryo, and suggested that the expression of VASH1 might not be limited to ECs [9]. Indeed, we could detect VASH1 mRNA in bone marrow hematopoietic stem cells [10]. Nevertheless, our immunohistochemical analysis preferentially detects VASH1 protein in ECs at the site of angiogenesis [4,8]. 

**Figure 1:**
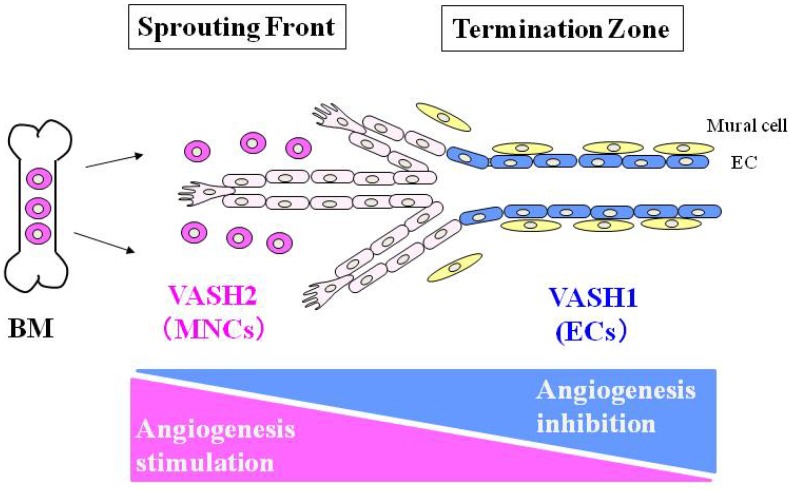
VASH1 is mainly expressed in ECs at the termination zone and halts angiogenesis. In contrast, VASH2 is mainly expressed in MNCs at the sprouting front and promotes angiogenesis. BM: bone marrow.

We further characterized the spatiotemporal expression and function of VASH1 during angiogenesis. Our analysis using the mouse subcutaneous angiogenesis model revealed that VASH1 is expressed not in ECs at the sprouting front but in newly formed blood vessels behind the sprouting front where angiogenesis terminates. In addition, *VASH1 (-/-)* mice contain numerous immature microvessels in the area where angiogenesis should be terminated (11). These results indicate that the principal function of endogenous VASH1 is to terminate angiogenesis (Figure 1). Importantly, newly formed immature microvessels in *VASH1 (-/-)* mice are functional, as indicated by blood flow [11].

We investigated the expression of VASH1 under various conditions accompanied by pathological angiogenesis. The presence of VASH1 in ECs is evident in various cancers, atherosclerotic lesions, age-dependent macular degeneration (AMD), diabetic retinopathy, and rheumatoid arthritis [12,13,14,15,16,17,18]. Even under pathological conditions, the extent of angiogenesis may vary in its natural course. Interestingly, patients with active AMD tend to have a lower VASH1-to-VEGF mRNA ratio, whereas those with the inactive disease have a higher VASH1-to-VEGF mRNA ratio [14]. As cancers contain complex lesions, where angiogenesis continues asynchronously and sprouting occurs randomly, it is difficult to dissect the expression profile of VASH1. Nonetheless, we showed that VASH1 is prevalent in tumor vessels of non-small cell lung cancers when they are associated with mural cells [17]. Thus, the spatiotemporal expression pattern of VASH1 is maintained even in tumor angiogenesis. Indeed, tumors inoculated into *VASH1 (-/-)* mice contain numerous immature vessels, resulting in a growth advantage of the tumors [17]. These observations suggest that VASH1 may regulate the course of angiogenesis under pathological conditions as well.

Exogenous VASH1 inhibits migration and proliferation of ECs, and inhibits angiogenesis [4]. One may ask how exogenous VASH1 protein can exhibit anti-angiogenic activity in the presence of endogenous VASH1. Our analysis elucidated that exogenous VASH1 exhibits little effect in the termination zone, where endogenous VASH1 is present, but effectively inhibits angiogenesis in the sprouting zone, where endogenous VASH1 is absent [11]. This observation further advocates the application of VASH1 to anti-angiogenic therapy. So far, the therapeutic effect of VASH1 has been shown in at least the following three different conditions; tumor angiogenesis, arterial adventitial angiogenesis associated with intimal thickening and ocular angiogenesis [4,12,19,20,21]. 

Peripheral lymphatic vessels are composed of a single layer of lymphatic ECs without mural cell coverage, and their function is to collect fluid lost from blood vessels and to maintain immune responses, lipid uptake, and tissue homeostasis. Recently, attention has focused on lymphangiogenesis, which is the formation of new lymphatic vessels, because it has been shown to be related to lymph node (LN) metastasis of cancers. Angiogenesis is counter-balanced by various endogenous inhibitors. However, little is known about endogenous inhibitors of lymphangiogenesis. Thrombospondin 1 (TSP1), an angiogenesis inhibitor, does not inhibit lymphangiogenesis [22]. Endostatin, another angiogenesis inhibitor, inhibits lymphangiogenesis and LN metastasis of certain tumors, but its effect on lymphangiogenesis is mediated via the down-regulation of VEGF-C, a principal lymphangiogenesis stimulator, in tumor cells [23,24]. We recently reevaluated the effect of VASH1 in the corneal micropocket assay, and observed its broad-spectrum anti-angiogenic as well as anti-lymphangiogenic activities [25]. In addition, we found that VASH1 inhibits tumor lymphangiogenesis and tumor LN metastasis [25]. To our knowledge, VASH1 is the first molecule intrinsic to the endothelium that exhibits such activities. 

## 3. Vasohibin-2 (VASH2)

By database searching, we isolated one gene homologous to *VASH1*, which we designated as *VASH2* [8]. Human VASH2 is composed of 355 amino acid residues, and the overall homology between human VASH1 and VASH2 is 52.5% at the amino acid level (Table 1). The gene for human *VASH2* is located on chromosome 1q32.3. So far, 11 exons for the *VASH2* gene have been shown in the database to form multiple transcripts for these paralogous genes owing to alternative splicing. The mouse *VASH2* gene is located at chromosome 1H6 spanning 31.48 kb. Investigating genes of the parasite *Schistosoma mansoni* by the comprehensive analysis, Venancia *et al*. found 14 different alternatively spliced forms of the *S. mansoni VASH2* gene [26]. These forms encode seven different protein isoforms including one with a complete C-terminal end, and other isoforms with shorter C-terminal portions. The significance of the VASH2 proteins expressed in *S. mansoni* is obscure at the moment. Nonetheless, this observation raises the issue of the evolution of *VASH* in the genome.

Because *VASH2* is related to *VASH1*, we examined whether or not VASH2 also participates in the regulation of angiogenesis. As described above, the expression of VASH1 is low in proliferating ECs at the sprouting front, but is high in non-proliferating ECs in the termination zone of angiogenesis [11]. In contrast, VASH2 is expressed preferentially in mononuclear cells (MNCs) that are mobilized from the bone marrow and infiltrate the sprouting front. In addition, angiogenesis in the *VASH2(-/-)* mice was deficient at the sprouting front [11]. These results indicate that VASH2 is expressed mainly in MNCs in the sprouting front and promotes angiogenesis (Figure 1). 

Delivery of VASH2 protein from the liver infected with adenovirus encoding *VASH2* gene sustained angiogenesis in the termination zone of wild-type mice, or normalized the deficient sprouting in the *VASH2(-/-)* mice [11]. Moreover, transient transfection of *VASH1* gene in ECs inhibited their proliferation, whereas that of *VASH2* gene stimulated it [11]. However, when recombinant VASH2 protein was locally applied in the cornea of wild-type mice, it inhibited angiogenesis [8]. These discrepancies may be explained by the context dependent activity of VASH2.

## 4. Concluding Remarks

The present mini-review has focused on the VASH family proteins, VASH1 and VASH2. Accordingly, their roles in the regulation of angiogenesis are distinct and perhaps contradictory. VASH1 is mainly expressed in ECs at the termination zone and halts angiogenesis, whereas VASH2 is mainly expressed in MNCs at the sprouting front and promotes angiogenesis (Figure 1). The question remains as to how these distinctive roles of VASH1 and VASH2 are controlled. In this context, the receptor for VASHs and their intracellular signaling pathways need to be determined. 

In terms of angiogenesis inhibitors, plural factors are expressed in ECs and control angiogenesis. For example, delta-like 4 (Dll4), a member of Notch ligand family, is expressed in tip cells, and controls the number of sprouts through Notch1 on stalk cells by lateral inhibition [27]. Thus, angiogenesis inhibitors intrinsic to ECs may orchestrate and control the entire processes of angiogenesis in a complementary manner.
